# Corticospinal Neurons in Macaque Ventral Premotor Cortex with Mirror Properties: A Potential Mechanism for Action Suppression?

**DOI:** 10.1016/j.neuron.2009.12.010

**Published:** 2009-12-24

**Authors:** Alexander Kraskov, Numa Dancause, Marsha M. Quallo, Samantha Shepherd, Roger N. Lemon

**Affiliations:** 1 Sobell Department of Motor Neuroscience and Movement Disorders, UCL Institute of Neurology, London WC1N 3BG, UK

**Keywords:** SYSNEURO

## Abstract

The discovery of “mirror neurons” in area F5 of the ventral premotor cortex has prompted many theories as to their possible function. However, the identity of mirror neurons remains unknown. Here, we investigated whether identified pyramidal tract neurons (PTNs) in area F5 of two adult macaques exhibited “mirror-like” activity. About half of the 64 PTNs tested showed significant modulation of their activity while monkeys observed precision grip of an object carried out by an experimenter, with somewhat fewer showing modulation during precision grip without an object or grasping concealed from the monkey. Therefore, mirror-like activity can be transmitted directly to the spinal cord via PTNs. A novel finding is that many PTNs (17/64) showed complete suppression of discharge during action observation, while firing actively when the monkey grasped food rewards. We speculate that this suppression of PTN discharge might be involved in the inhibition of self-movement during action observation.

## Introduction

The first demonstration of mirror neurons in the macaque brain (di Pellegrino et al., 1992; Gallese et al., 1996; Rizzolatti et al., 1996a) showed that they became active both when the monkey performed a given action and when the monkey observed a similar action performed by the experimenter. Mirror neurons were found to constitute a significant fraction (17%) of cells recorded in the rostral division of the ventral premotor cortex (area F5). Further investigations have demonstrated that mirror neurons can fire when a grasping action is performed just out of sight of the monkey (Umiltà et al., 2001) and can differentiate whether the action is carried out in the peripersonal or extrapersonal space of the monkey (Caggiano et al., 2009). Some F5 mirror neurons are particularly responsive to orofacial movements related to eating and communication (Ferrari et al., 2003), while others respond to actions performed with tools (Ferrari et al., 2005) or to sounds characteristic of particular actions (Keysers et al., 2003; Kohler et al., 2002). Neurons with “mirror-like” activity are not confined to F5 but are also found in the inferior parietal lobule (Fogassi et al., 2005; Rozzi et al., 2008). Their presence may be even more widespread (Cisek and Kalaska, 2004; Tkach et al., 2007), suggesting that the mirror neuron system involves a network of areas.

A number of noninvasive studies have demonstrated a similar mirror neuron system in the human brain (Grafton et al., 1996; Rizzolatti et al., 1996b), which has been implicated in a variety of cognitive functions, including understanding the nature and meaning of actions, a wider role in imitation, speech, and emotion (Fabbri-Destro and Rizzolatti, 2008; Iacoboni, 2009), as well as in a spectrum of neurological disorders (Rizzolatti et al., 2009).

However, despite the diversity of exciting potential roles for such a system, we still know very little about the identity of the mirror neurons themselves. Because extracellular recording in monkey cortex is inherently biased toward large neurons, it is likely that all mirror neurons are pyramidal neurons rather than cortical interneurons. However, it is unknown whether mirror neurons are involved in processing of afferent inputs or represent outputs to other motor structures. Because PMv contributes to the corticospinal tract (Dum and Strick, 1991), it is critical to investigate whether pyramidal tract neurons (PTNs) within area F5 show mirror-like activity.

## Results

This study was carried out in two macaque monkeys trained to perform both a precision grip task (Baker et al., 1999) and a tool-use task that involved use of a rake to retrieve food rewards (Ishibashi et al., 2000; Quallo et al., 2009; Experimental Procedures). However, this paper reports only studies involving grasping actions and action observation by these monkeys and is entirely focused on fully analyzed recordings from 64 identified PTNs selected for recording on the basis of their antidromic activation from the pyramidal tract (PT). Most of the PTNs (53) were recorded in a monkey (M43) in which we also made simultaneous EMG recordings from nine arm, hand, and digit muscles; another 11 PTNs were recorded in the second monkey (M41). All PTNs were recorded in the rostral division (area F5; Matelli and Luppino, 1996) of the ventral premotor cortex (PMv) in the right hemisphere (Supplemental Data, Figure S1). Area F5 was characterized by brisk neuronal activity for grasp with either the contra- or ipsilateral hand and by the presence of contralateral digit movements evoked by ICMS at thresholds >15 μA at recording sites.

### F5 PTNs

**Figure 1:**
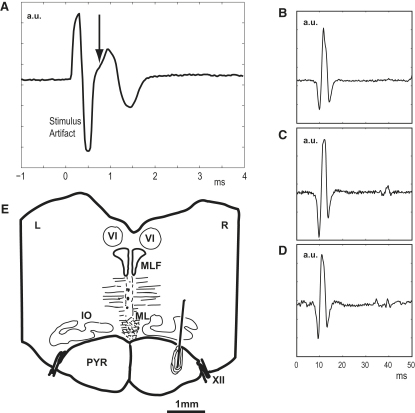
Stimulation of the Pyramidal Tract (A) Antidromic volley, recorded during surgery in M41, from the cortical surface of M1 to stimulation of the ipsilateral PT (bipolar stimulation through two implanted electrodes 5 mm apart in PT, single biphasic shock, 200 μA). Vertical arrow indicates beginning of antidromic response (∼0.7 ms from PT stimulus onset at zero time). Average of 100 sweeps. (B–D) Short-latency EMG response in contralateral thenar muscles to PT stimulation (single bipolar shock 250 μA). These were recorded in M43 awake at the beginning (C), middle (D), and end (E) of the experimental period. Averages of 50 sweeps. (E) Transverse section through brainstem of M41, showing location of tip of posterior PT electrode on right (R) side and surrounding gliosis. PYR, pyramidal tract; IO, inferior olive; ML, medial lemniscus; MLF, medial longitudinal fasciculus; VI, abducens nucleus; XII, hypoglossal nerve.

Most PTNs (75%) were recorded 1–3 mm from electrode entry and were probably located on the convexity of the ventral precentral gyrus. The database contained only those PTNs that satisfied strict criteria for antidromic activation, including the collision test (see Experimental Procedures), and whose spikes could be reliably discriminated throughout a range of tests designed to evoke mirror-like activity (Experimental Procedures). The implanted electrodes used to identify these PTNs were confirmed to be located in the ipsilateral pyramidal tract (PT) by a number of electrophysiological and histological tests (Experimental Procedures, Figure 1).

### “Classic” Mirror Neurons

**Figure 2:**
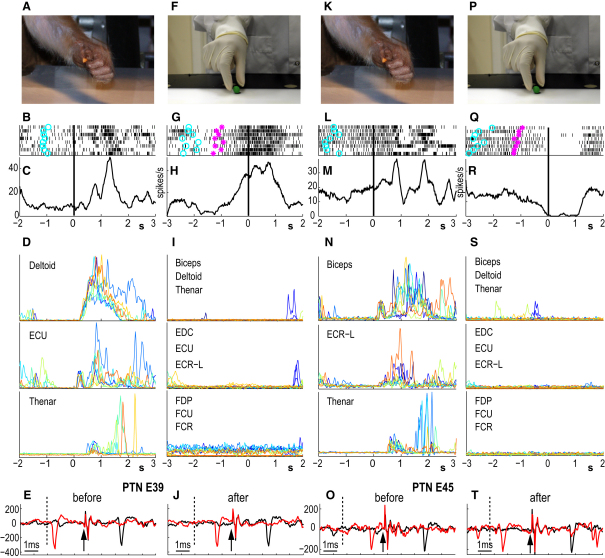
Examples of Two Different Types of Response in F5 PTNs: One Activated during Action Observation and the Other Suppressed (A and K) Photo of monkey grasping a piece of food in a precision grip; (F and P) photo of experimenter grasping a piece of food in precision grip; (B and L) raster plots for two PTNs during self-grasp aligned to cue for onset of reach-to-grasp movement (indicated by black vertical lines); data from ten successive trials are shown. Note that there were several bursts of activity in both PTNs, associated with initial grasp of reward and then release of food at the mouth, with each burst associated with peaks of EMG activity in distal muscles, such as thenar (see traces in D and N). (G and Q) raster plots for two PTNs during ten trials of mirror testing (precision grip with object) aligned to the moment of contact of the experimenter's hand with the target object (indicated by black vertical lines). Light-blue circles on each trial indicate beginning of baseline interval for each trial (experimenter's hand motionless in full view of monkey), and magenta asterisks indicate beginning of experimenter's movement toward the object. (C, H, M, and R) Average firing rates based on rasters above (spikes/s); (D and N) superimposed records of EMG activity from three muscles (D: deltoid, ECU, and thenar; N: biceps, ECR-L, and thenar) during the same ten self-grasp trials as in (B) and (L), respectively; (I and S) superimposed records of EMG activity from all nine muscles during the same ten mirror testing trials shown in (G) and (Q), respectively. Muscles are divided into three groups of three records. EMG activity from the same trials are in same color; all EMGs are autoscaled to the maximum activity during self-grasp. Note almost complete absence of EMG activity during mirror testing (I and S). (E, J, O, and T) Antidromic responses from PTNs in response to PT stimulation, onset of PT stimulus is indicated by arrows, black curves are averages over tens of trials, antidromic spikes had constant latency throughout (facilitation cell [E and J] 2.4 ms, suppression cell [O and T] 2.8 ms) before (E and O) and after (J and T) mirror testing; red curves show collisions when a spontaneous spike appeared after the collision interval (indicated by dotted line): the antidromic spike was collided and absent from the record.

The main findings are illustrated by two F5 PTNs (Figure 2) with completely different patterns of activity. The first PTN, shown in Figures 2A–2J, showed bursts of activity when the monkey reached out and used its contralateral hand to grasp a small piece of food placed in front of it (see photo Figure 2A). Rasters of discharge for a block of grasp trials (Figure 2B) and averaged discharge (Figure 2C) were aligned to the cue for the monkey to begin its reach-to-grasp movement, which was accompanied by bursts of EMG activity in arm (deltoid), wrist (extensor carpi ulnaris, ECU), and digit (thenar) muscles (Figure 2D). The mirror-like activity of this PTN is shown in Figures 2F–2I. The experimenter, sitting directly in front of the monkey, started each trial with her right hand motionless on a touch-sensitive pad. After a short baseline period, she released her hand from the pad (magenta asterisks in Figure 2G) and approached a piece of food previously placed in an “action observation area” that was in the monkey's midline and just beyond its reach. The monkey sat calmly throughout this process and made no hand or arm movements, as evidenced by the complete absence of EMG activity in all recorded muscles during the period of action observation (−750 to +750 ms). Figure 2I shows all superimposed trials of EMG recordings from nine arm, wrist, and digit muscles; all recordings were essentially flat. As the experimenter's hand neared the food, the PTN steadily increased its firing rate, peaking after the experimenter's hand touched the food target, and grasped it in a precision grip. This moment (indicated by the black line at time 0) was detected by a sensor located within the observation area responding to a small magnet attached to the tip of the experimenter's finger (Experimental Procedures). Note the reproducible pattern of discharge in the raster plots for the block of action observation trials. Note also that during action observation, the PTN fired with maximum rates similar to those observed during the monkey's own grasping movement (Figure 2C). Antidromic stimulation and use of the collision test (Baker et al., 1999) confirmed that the same PTN was recorded throughout the periods of active grasp (Figure 2E) and action observation (Figure 2J).

### Suppression Mirror Neurons

A quite different pattern was found for other PTNs, an example of which is shown in Figures 2K–2T. In this case, although the PTN again showed increased bursts of activity as the monkey reached and grasped the food reward (Figures 2L and 2M), its background discharge was completely suppressed during action observation (Figures 2Q and 2R). Once again, this suppression of activity was highly reproducible from trial to trial (Figure 2Q). Another example of suppression-type PTNs is shown in Figure S5.

### Population Analysis

Of the 64 PTNs analyzed, 31 (49%) showed statistically significant modulation in their discharge in the 1.5 s period centered around the sensor signal (Experimental Procedures). Of these PTNs, 14/64 (22%) were of the facilitation type (cf. Figures 2G and 2H) while 17/64 (27%) showed suppression (cf. Figures 2Q and 2R). Both types were recorded in both monkeys. For the 53 PTNs recorded in monkey M43, we analyzed EMG and excluded a total of 15 PTNs that were recorded in sessions where there was significant modulation of EMG activity during action observation (see Experimental Procedures and Supplemental Data). Of these remaining 48 PTNs, 11 (23%) were of the facilitation type, while 14 (29%) showed suppression.

**Figure 3:**
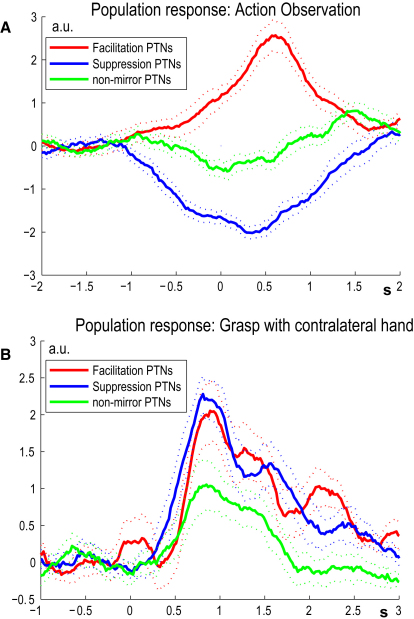
Population Summary of Activity in F5 PTNs (A) Population average of PTN firing rates during action observation of precision grip with an object and (B) for active grasp of a food reward by the monkey with its contralateral hand. Data plotted separately for mirror PTNs, which showed facilitation (n = 11, red) and suppression effects (n = 14, blue) during action observation, or no effect (non-mirror PTNs, n = 23, green). Dotted lines indicate standard error of the mean. Alignment at time zero in (A) is to the touch of magnetic sensor by experimenter and in (B) to the cue for the monkey to begin its reach-to-grasp movement. Note that both facilitation and suppression types of PTN increased their discharge during active grasp, with the increase beginning shortly after the cue and associated with the first burst of EMG activity in distal muscles. Firing rate for each PTN is in arbitrary units (a.u.): the rate was first normalized to unitary standard deviation over −2 to 2.5 s period, and then shifted to have average baseline (between −2 and −1) equal to zero. Data plotted are for PTNs recorded during sessions with no significant EMG activity during action observation.

The population responses of both types of F5 mirror-like PTNs during action observation, aligned to the sensor signal, are presented in Figure 3A. Facilitation (red line) and suppression (blue) types showed changes in firing rate around the time that the experimenter began the movement (−1 s; magenta asterisks in Figures 2G and 2Q). The facilitation type showed the classical pattern of mirror-like activity with facilitation during both action observation (Figure 3A) and grasp (Figure 3B). The suppression type showed a strong and protracted suppression of discharge during action observation (Figure 3A), but, like the PTN in Figures 2L and 2M, were facilitated during active grasp (Figure 3B), with a similar time course to that of the facilitation type of mirror PTN. Indeed, 14/17 (83%) suppression-type PTNs, like most F5 neurons, showed significantly increased discharge when the monkey actively grasped the food with the contralateral hand. Of course, detection of a suppression response during action observation required the PTN to have some ongoing, background discharge activity, and it is known that PTNs generally have higher rates than other classes of cortical neuron (Baker et al., 2001; Merchant et al., 2008). We found that the mean background firing rate for PTNs showing suppression (14.8 ± 6.0 sp/s) was significantly higher than that of PTNs showing facilitation (6.2 ± 6.1 sp/s; p < 0.001, Mann-Whitney U test). The average firing rate of F5 PTNs that did not exhibit any mirror-like activity (Figure 3A, green line) showed only a slight deviation from baseline throughout whole observation period. During active grasp (Figure 3B, green), they were activated to a somewhat less marked degree than mirror PTNs.

### “Mirror-like” Modulation in Different Contexts

**Figure 4:**
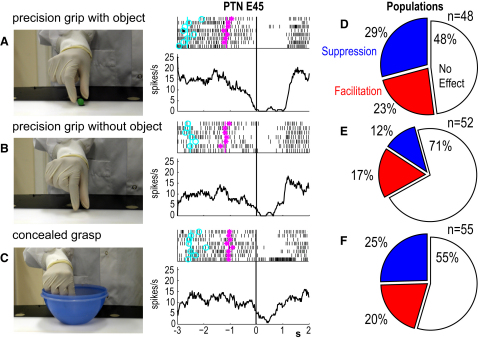
PTN Activity during Observation of Grasp in Different Contexts (A–C) Raster plots and average firing rates during action observation of grasps carried out under three different conditions. The photographs show the actions as seen by the monkey. In (A), the experimenter performed a precision grip with an object present, in (B), a precision grip without an object being present, and in (C), the grasp was concealed from the monkey by the opaque walls of the food bowl. Background discharge in this F5 PTN was suppressed in all three conditions. Rasters and average firing rates aligned to the touch of magnetic sensor by experimenter's finger tip. Other notations as in Figure 2. (D–F) Percentage of PTNs recorded in EMG-free sessions (see Experimental Procedures), which exhibited significant “mirror-like” activity (p < 0.05, a Mann-Whitney U test) during the three grasp conditions. PTNs facilitated or suppressed during action observation in red and blue, respectively. PTNs with no mirror activity shown in unfilled segment.

Most F5 PTNs exhibited mirror-like modulation of discharge during observation of grasps carried out in different contexts by the experimenter (Experimental Procedures). In the example shown in Figures 4A–4C, the PTN's ongoing discharge was completely suppressed when the monkey observed the experimenter performing precision grip of food (Figure 4A), but also when this same precision grip was carried out without the food being present (Figure 4B). In other words, this PTN's discharge was also suppressed when an “intransitive” action, with no apparent goal, was carried out by the experimenter. Discharge suppression also occurred when the experimenter reached into a small food bowl; her grasp within the bowl was concealed from the monkey by the bowl's opaque sides (Figure 4C).

73% of PTNs that were modulated during observation of precision grip with an object were modulated during precision grip without an object, and 64% were modulated during concealed grasp. The pie charts in Figures 4D–4F show the proportion of PTNs with mirror-like properties that were detected in action observation sessions free of EMG contamination. Similar proportions of PTNs showed significant modulation during action observation of precision grip with an object (52%; Figure 4D) and concealed grasp (45%; Figure 4F), but were less common during precision grip without an object (29%; Figure 4E). In all three contexts, PTNs showing suppression of discharge were at least as common as those showing facilitation of their discharge. Suppression of discharge was also seen in some of these PTNs during other types of action, such as advancing of the experimenter's hand toward a reward and covering the reward with the outstretched hand, but not grasping it (Supplemental Data).

### Antidromic Latencies of PTNs

**Figure 5:**
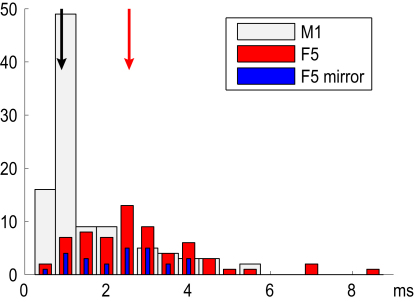
Antidromic Latencies of F5 and M1 PTNs Distribution of antidromic latencies (ADLs) for PTNs in F5 (n = 64 in red; mean ADL 2.8 ± 1.5 ms) and in M1 (n = 100, open columns; mean ADL 1.5 ± 1.2 ms) and recorded in M41 and M43. The subset of F5 PTNs that showed “mirror-like” activity is shown in blue. The red vertical arrow indicates median value of ADLs for F5 PTNs (2.6 ms) and the black arrow for M1 PTNs (0.9 ms). These two populations were significantly different (p < 1e–6, Mann-Whitney U test). We did not find any significant difference between ADLs of mirror and non-mirror PTNs.

Figure 5 shows that F5 PTNs (red bars) had relatively long antidromic latencies (median ADL 2.6 ms, indicated by red arrow) and clearly belonged to a slowly conducting population significantly slower than the fast-conducting M1 PTNs (open columns; median ADL 0.9 ms, indicated by black arrow) encountered in the M1 hand area of the same monkeys (Figure S1). Although the majority of M1 PTNs had ADLs close to 1 ms, we also recorded some slow M1 PTNs. The distribution of F5 PTNs was not as skewed as in M1 and was more symmetrical around the median. The subset of F5 PTNs that showed mirror-like activity is shown with blue bars. Their ADLs were not significantly different from the subset without mirror-like activity.

## Discussion

We have demonstrated that within area F5 of ventral premotor cortex mirror-like activity is present in output neurons that project through the pyramidal tract and in all likelihood to the spinal cord (Humphrey and Corrie, 1978; Lemon, 2008). Thus, activity within the mirror-neuron system is communicated directly to other regions of the motor network, including its subcortical components, and will probably have functional consequences for these components. The F5 PTNs we recorded were found in the same region of area F5 as in the original report by Gallese et al. (1996) and were mostly located on the convexity of the gyrus, as predicted from retrograde labeling of corticospinal neurons (He et al., 1993). A significant proportion of these PTNs were activated during observation of grasping actions in different contexts, including intransitive precision grip and grasp that was concealed from the monkey. We do not consider that the mirror-like activity in PTNs that were facilitated during action observation resulted from covert movement by the monkey. Our data show unequivocally that mirror-like activity can be evoked without any concomitant EMG activity (Figure 2).

A novel finding is the description of F5 PTNs with a steady resting discharge that was completely suppressed during the mirror tests. To date, all published records of mirror neurons are characterized by a very low or absent background discharge that changes to a brisk increase in firing rate during both action observation and the monkey's own grasp (Caggiano et al., 2009; Fogassi et al., 2005; Gallese et al., 1996; Rozzi et al., 2008). There have been no published examples of firing suppression in F5 (but see Supplementary Materials of Caggiano et al., 2009). The suppression type of PTN was as prevalent in our sample of F5 PTNs as the facilitation type (56% versus 44%). Preliminary comparison with recordings of other neurons in the same monkeys and sessions demonstrates that suppression of activity may be significantly more common among PTNs in F5 compared with unidentified neurons in the same area. Interestingly, these suppression PTNs, unlike classical mirror neurons, provide an unambiguous signal as to whether the current state involves action observation (suppressed discharge) or active grasp (elevated discharge; see Figure 3). The two classes of facilitation and suppression mirror neurons may operate independently or be part of the same functional circuit.

The presence of two different patterns of mirror-like activity in PTNs provides a possible explanation for contrasting changes in the excitability of the human motor system during action observation. Thus, facilitation mirror PTNs might explain the “motor resonance” phenomenon in which the excitability of hand motoneurons in an observer mirrors or resonates with that of the actor (Cattaneo et al., 2009; Fadiga et al., 1995; Montagna et al., 2005; Strafella and Paus, 2000). Other studies have demonstrated dynamic and specific mechanisms that may serve to prevent such resonance being elaborated into overt movements (Baldissera et al., 2001). We speculate that such changes could result from a disfacilitation of motoneurons caused by suppression of activity in PTNs during action observation.

It should be stressed that although we suggest that these PTNs might be concerned with suppression of self-movement during action observation, we think of this as a suppression mechanism that might allow the withholding of a number of hand actions, rather than suppression of one particular act, for example the suppression of imitation. Indeed, many F5 PTNs were suppressed during observation of grasping actions in a variety of contexts (Figure 4). A role for macaque PTNs in imitation, or its suppression, seems unlikely, since it is known that adult macaque monkeys have a limited capacity for motor imitation (Ferrari et al., 2006; Visalberghi and Fragaszy, 2002).

In macaques, F5 contributes a relatively small proportion of the total corticospinal output from the frontal lobe, around 4% (Dum and Strick, 1991). Most of the F5 PTNs we sampled had rather slowly conducting axons and are obviously different from the large, fast PTNs that characterize the M1 output (Figure 5). Only a minority of F5 PTNs terminate in the lower cervical motor nuclei innervating digit muscles (He et al., 1993). In terms of facilitatory actions, it has not yet been shown that F5 makes any contribution to direct cortico-motoneuronal excitatory projection (Lemon, 2008; Rathelot and Strick, 2006, 2009) and F5 PTNs exert only weak facilitatory effects on digit muscles (Boudrias et al., 2009; Cerri et al., 2003). Motor effects elicited by ICMS, similar to those found at F5 recording sites in this study, may be largely mediated through cortico-cortical interactions with M1 (Schmidlin et al., 2008; Shimazu et al., 2004).

In terms of inhibitory actions, F5 PTNs could exert a net inhibitory effect via segmental inhibitory circuits (Cheney et al., 1985; Prut and Fetz, 1999) or feedforward inhibition of propriospinal systems (Isa et al., 2007) at more rostral cervical levels, which is their principal target (He et al., 1993). They may also be part of the cortico-cortical circuit that exerts suppression of M1 outputs as part of the active shaping of the hand for grasp (Prabhu et al., 2009).

We consider it unlikely that the mirror-like activity in PTNs that were facilitated during action observation resulted from covert movement on the part of the monkey. This concern was already recognized by Gallese et al. (1996) but was refuted based on quiescent EMG activity in hand and mouth muscles during action observation. However, in that study, EMGs were examined in separate sessions from those in which the neuronal activity was recorded. The simultaneous EMG evidence we collected from M43 supported the conclusion that the activity of the PTNs evoked during action observation was not due to covert movement on the part of the monkey (Figure 2). This was further supported by other lines of evidence that showed that only a proportion PTNs that were active for the monkey's own grasping movement showed mirror-like activity and that it was possible to record simultaneously from pairs of PTNs, both of which discharged during active grasp but only one of which showed mirror properties (Supplemental Data, Figure S4).

Finally, a striking finding of this study is the relatively high proportion of F5 PTNs tested that showed mirror-like activity. It is possible that training on the rake task (Ishibashi et al., 2000), which involved a large amount of interaction with the experimenter and which might have influenced the monkeys' cognitive ability to associate observation with action (Iriki and Sakura, 2008), may have influenced this result.

The present findings give new insights into the potential functions of the mirror neuron system. In addition to the wide range of functions suggested in the literature, including the understanding and imitation of actions carried out by others, and the prediction of the outcome of such actions (Fabbri-Destro and Rizzolatti, 2008; Rizzolatti et al., 2009), the mirror neuron system might also be involved in the inhibition of unwanted self-movement during action observation. This last suggestion will need to be confirmed by future studies that examine the responses of F5 PTNs whose discharge is suppressed by action observation, during performance of other tasks requiring the withholding of a motor response.

## Experimental Procedures

All experimental procedures were approved by the Local Ethical Procedures committee and carried out in accordance with the UK Animals (Scientific Procedures) Act. Experiments were performed on two adult purpose-bred Rhesus (*M. mulatta*) monkeys, (M41, male 8.0 kg and M43, female 5.5 kg).

### Behavioral Tasks

Monkeys were trained to perform two active tasks, both with the left hand. The first was a precision grip task in which the monkey was required to use its index finger and thumb to squeeze two spring-loaded levers into an electronically defined target (Baker et al., 1999). The levers were mounted in a manipulandum positioned just in front of the monkey. Monkeys were trained to move each lever independently into the target zone and hold it there for around 1 s; each successful trial was rewarded by the experimenter passing a small food reward to the monkey (fruit, nuts, or pulses). The second task involved the monkey using a tool (Ishibashi et al., 2000). A flat white table (62 cm deep by 68 cm wide) was placed in front of the monkey. The monkey used a rake to retrieve a small food reward placed by the experimenter on the table just beyond the monkey's reach, i.e., in its “extrapersonal” space. Monkeys were trained on these tasks for several months and reached a steady level of performance before recording began (M41, 7 months; M43, 9 months). They were not exposed to the mirror testing protocol until after recording began.

### Mirror Testing

The mirror test procedure comprised of five different tests, each with ten trials. All five of these tests were carried out in both monkeys, in blocks of ten trials on the table in front of the monkey. Each trial began with the experimenter's right hand resting on a central homepad (Figure S2: HPP). About 1.5 s later, a tone sounded, which cued the experimenter to release the homepad (Figure S2: HPR) and begin the specific test. The experimenter wore a glove on the right hand, and this glove contained a small magnet at the tip of the finger. As the experimenter approached the action observation area of the table, which was in the monkey's midline and just beyond its reach, a magnetic sensor embedded in the table at the center of this area was activated and generated a sensor pulse (Figure S2: SP). The timing of the homepad signal and of this sensor pulse were recorded along with neuronal and EMG data. Tests were repeated once every 4–5 s.

#### Mirror Test 1: Precision Grip with Object (see Figure 4A)

A small piece of food was placed above the central sensor. After releasing the homepad, the experimenter slowly approached the food and grasped it in a precision grip between the tips of the thumb and index finger. The monkey was rewarded after every fifth trial.

#### Mirror Test 2: Precision Grip without Object (see Figure 4B)

After releasing the homepad, the experimenter slowly approached the central sensor and pantomimed the movement of picking up the food in a precision grip. The monkey was rewarded after every fifth trial.

#### Mirror Test 3: Concealed Grasp (see Figure 4C)

A bowl full of small pieces of fruit, which has been used throughout the training of both monkeys, was positioned above the central sensor. After releasing the homepad, the experimenter put her right hand into the bowl and retrieved a piece of food, although the grasp itself was concealed from the monkey because of the opaque walls of the bowl.

#### Test 4: Monkey Grasps with Contralateral Hand (see Figure 2A)

The experimenter took a small piece of food and placed her hand on the homepad. As she released the homepad she placed the food reward on the table to the monkey's left where it could easily reach and grasp with its left (contralateral) hand. Her release of the homepad cued the monkey's reach-to-grasp movement.

#### Test 5: Monkey Grasps with Ipsilateral Hand

As for test 4, but food placed to right of monkey and grasped with the ipsilateral hand.

In tests 1–4, the experimenter gently restrained the monkey's right hand (ipsilateral to recording); in test 5, the experimenter held the monkey's left (contralateral) hand.

### Surgical Preparations and Electrophysiological Recordings

Under deep general anesthesia, a headpiece was surgically implanted to allow head restraint (Lemon, 1984). During a second surgery (M43 only), we implanted chronic EMG patch electrodes in nine arm, hand, and digit muscles (Brochier et al., 2004). During a final surgery, a single 20 × 10 mm recording chamber was mounted so as to give access to the inferior limb of the arcuate sulcus (for F5 hand area recordings) and to the middle third of the central sulcus (for M1 hand area recordings) in the right hemisphere (Figure S1 shows the location for M43). The extent of the craniotomy was based on central and arcuate sulci locations obtained from previous structural MRI scan (Baker et al., 1999). During surgery, sulcal locations were measured sterotaxically through the dura and checked against MRI-derived measurements. During the same surgery, a pair of fine (240 μm shank diameter) tungsten stimulating electrodes were chronically implanted in the right medullary pyramid for subsequent antidromic identification of pyramidal tract neurons (Olivier et al., 2001). Electrodes had tip impedances of 20–30 kΩ. The stereotaxic location of the electrodes was anterior +2, lateral 0.5, and posterior −3, lateral 0.5 for anterior and posterior electrodes, respectively. The final depth of each PT electrode was determined from the lowest threshold point (usually <20 μA) for activation of the short-latency antidromic volley recorded through the dura from the ipsilateral M1 (see Figure 1A). Subsequent testing of these electrodes in the awake monkey confirmed that single PT shocks of 150–200 μA evoked short-latency EMG responses in intrinsic hand muscles (see Figure 1B; Olivier et al., 2001), which remained consistent throughout the recording period (Figures 1B–1D). Finally, histological analysis in M41 confirmed the accurate location of the electrode tip in the PT (see Figure 1E).

At the end of the experiment in M41, the monkey was killed by an overdose of pentobarbitone (50 mg kg^−1^ i.p. Euthanal; Rhone Merieux) and perfused through the heart. The cortex and brain stem were photographed and removed for histological analysis. The implanted electrode tips were confirmed to be in the PT (Figure 1E). M43 is still alive.

Cortical recordings were made using a Thomas Recording 7-channel drive. The stereotaxic position of the tip of the drive was calculated by triangulation using fiducial markers on the chamber lid. The drive carried 3–5 glass-insulated platinum electrodes (diameter, 80 μm) with an interelectrode spacing of 300 μm. The drive was controlled through a networked system that allowed several experimenters (usually two to three) to advance each electrode independently (Baker et al., 1999).

In M43, EMG was recorded with chronic subcutaneous electrodes from the following nine muscles: anterior deltoid, biceps brachii, extensor and flexor carpi ulnaris (ECU, FCU), extensor carpi radialis (ECR-L), flexor carpi radialis (FCR), flexor digitorum profundus (FDP) extensor digitorum communis (EDC), and thenar muscles. We used the technique described in Brochier et al. (2004); correct placement of the EMG electrodes was confirmed by stimulation through the recording leads that were terminated in a multipin socket on the animal's back.

Following preamplification (×20, Thomas Recording drive), the signals from each electrode were further amplified (typically ×500 or ×1000) and band-pass filtered (0.3–10 kHz). Data were acquired using A/D card (PCI-6071E, National Instruments) at 25 kHz sampling rate and were recorded together with EMG activity and experimental time events including home pad and sensor signals. Wherever possible, recordings were made simultaneously from several PTNs.

When a full set of data had been recorded for each group of PTNs sampled, an isolated stimulator (Neurolog NL800 stimulus isolator, Digitimer, UK) was used to deliver trains of repetitive intracortical microstimulation (ICMS) through each recording electrode (13 pulses at 333 Hz, intensity typically up to 50–60 μA, duty cycle 0.5 Hz), in order to characterize the output effects from the recording site. After the electrodes had been withdrawn, the exposed dura was treated for 5 min with the antimitotic compound 5-flurouracil, to counteract dural scarring (Spinks et al., 2003), before the chamber was thoroughly washed with sterile saline and then sealed.

### Identification of PTNs

During each recording session, we searched for PTNs by looking for antidromic responses having an invariant latency (jitter < 0.1 ms) to each PT shock. A search stimulus of 250–300 μA (biphasic pulse, each phase 0.2 ms) was applied to the PT electrodes. Once a PTN had been identified and well-isolated, its antidromic latency (ADL) was measured from the beginning of the stimulus artifact to the first negative peak in the action potential. Spontaneous, orthodromic spikes from the PTN were discriminated online using a software-based double time-amplitude discriminator. Responses evoked from the PT were then confirmed to be antidromic by using these spontaneous spikes to collide the antidromic spikes (Baker et al., 1999; Lemon, 1984); see Figures 2E, 2J, 2O, and 2T). Figure 5 shows the distribution of ADLs for the PTNs recorded in F5 (red bars; blue bars are PTNs with mirror-like activity). The distribution is dominated by PTNs with long ADLs (>2 ms) and is significantly different from that for M1 PTNs (open bars) recorded in the same monkeys.

While we searched for PTNs, the monkey sat quietly and was not performing a task or being tested for action observation. Thus, our sample is completely unbiased in terms of the unit's natural activity which was not tested until stable recordings had been achieved.

### Analysis of PTNs

PTNs were detected and clustered using modified *Wave_clus* software (Quiroga et al., 2004). We used an extended set of features that included not only wavelet coefficients but also first three principal components. Spike shapes of PTNs obtained after clustering were checked against shapes of spikes that spontaneously collided antidromic spikes during PT stimulation before and after mirror testing (see e.g., Figures 2E, 2J, 2O, and 2T). During spike detection, a very short (200 μs) “dead” time between two consecutive spike events was used, which allowed detection of different units that fired close together in time. For bursting units, clusters with minimum 1 ms interspike interval were accepted; for other units, a minimum interspike interval of 2 ms was set.

To test whether a cell showed any modulation during action observation, we compared baseline activity (750 ms before home pad release) and activity during mirror movement (750 ms before and after the sensor signal) using a Mann-Whitney U test (p < 0.05).

Only cells that showed statistically significant (p < 0.05) modulation in firing rate during mirror tests were retained to construct the population averages of PTNs with mirror-like activity (Figure 3). The time course of each PTN's discharge was normalized to unit standard deviation and, for illustration purposes (Figure 3), shifted to have zero mean value over baseline interval (here −2 to −1 s).

### EMG Analysis

To confirm that mirror activity in PTNs was not due to covert movements, in monkey M43, we carefully checked data from every session for any signs of modulation of EMG activity during action observation. We analyzed EMGs from all nine muscles. EMGs were first low-pass filtered (second order Butterworth, 10 Hz cut off frequency) and the resulting signal was analyzed using the same time intervals and statistical test as used for analysis of PTN activity. For each trial, an average baseline EMG activity was estimated as a median over 750 ms interval before the end of the HP signal. It was then compared, using a Mann-Whitney U test, with the median EMG amplitude estimated over 1.5 s centered on the sensor signal. If the difference was found to be significant, we removed outlier trials from the tails of the distribution. We eliminated the most distant from the median trial and then repeated the statistical comparison. This procedure was repeated until the difference was not significant (p > 0.05). Elimination of the trials was performed for each muscle independently. If the difference still remained significant for at least one muscle or if the total number of remaining trials over all muscles was smaller than seven, we discarded all PTNs from that recording session from further consideration (see Figure S3 as an example of a discarded session). After completion of this analysis in M43, 15 PTNs were excluded. Only six of them showed mirror-like activity; three PTNs showed facilitation and three suppression. The F5 PTNs illustrated in the main text (Figures 2 and 4) all showed significant modulation during action observation that was free of any concomitant EMG activity (cf. Figure 2I and 2S). The proportion of PTNs with mirror activity in the sample taken from M43 before and after EMG control was comparable (51% before and 54% after).
