# Supplementary material for: Corticospinal Neurons in Macaque Ventral Premotor Cortex with Mirror Properties: A Potential Mechanism for Action Suppression?
Source: Neuron. 2009 Dec 24;64(6):922–30. doi: 10.1016/j.neuron.2009.12.010 (PMC2862290; doi:10.1016/j.neuron.2009.12.010)
Supplement: Document S1. Supplemental Data [file mmc1.pdf]

Neuron, Volume 64

Supplemental Data

Corticospinal Neurons in Macaque Ventral Premotor Cortex with Mirror Properties: A Potential Mechanism for Action Suppression?

A. Kraskov, N. Dancause, M. Quallo, S. Shepherd, and R.N. Lemon

Control experiments: Mirror-like responses are not due to covert actions on the part of the monkey

Since most area F5 neurons, including PTNs, are movement-related (Kakei et al., 2001; Kurata, 1993; Kurata and Hoshi, 2002; Rizzolatti et al., 1988; Umilta et al., 2007), it is possible that facilitation of their discharge during action observation was the result of small or covert movements made by the monkey. Overall this seems unlikely, since most of the PTNs sampled showed significant modulation in their activity during the monkey's own grasping action (53/64; 83%), while only 14 (22%) showed significant excitatory mirror-like activity. Nevertheless, in a few recording sessions, we noticed sporadic trials in which the experimenter's actions did indeed prompt the monkey to make small movements and these were accompanied by bursts of EMG activity recorded from contralateral arm and hand muscles; these bursts were of a similar magnitude to those seen during active grasp by the monkey (see Fig. S3).

Two further findings reinforced the conclusion that mirror-like activity in F5 PTNs did not result from the monkey's own movements. First, in monkey M43, although EMG recording allowed us to exclude possible confounds due to movements of the contralateral hand, we could not exclude PTN activity due to covert movements of the ipsilateral hand, or indeed other body parts, including the mouth, for which no EMG was available. We tested this in a number of ways.

For example, a few PTNs were encountered which did not modulate their discharge at all when the monkey grasped with its ipsilateral hand (example PTN shown in Fig. S4A,B). Two out of three PTNs of this kind showed clear 'mirror-like' facilitation of their discharge (see Fig. S4C) which therefore could not have resulted from covert movements of the ipsilateral hand.

Second, we recorded a small number of F5 neurons whose activity was related to licking and chewing movements. Covert movements of this kind might be expected to occur during action observation (Cattaneo et al., 2007), when food was being grasped by the experimenter. However, none of these mouth-related neurons showed significant modulation of discharge during action observation.

Finally, because we made simultaneous, multiple-electrode recordings of PTNs and other unidentified neurons we could check whether 'mirror-like' modulation was due to other non-specific changes in the monkey's attention or anticipation. We could demonstrate that while the monkey watched a specific set of actions carried out by the experimenter, some PTNs showed 'mirror-like' activity while others did not. An example is shown in Fig. S4D and E. One PTN showed pronounced mirror-like facilitation (Fig. S4D), while another, recorded on a separate electrode only a few 100 microns away during the same set of trials, showed no modulation (Fig. S4E).

PTNs respond to grasp not kinematics of the approach movement

During tests for 'mirror-like' activity, all the actions were carried out slowly and deliberately in front of the monkey. We investigated whether PTNs modulated their discharge while the monkey observed the experimenter approaching and beginning to grasp in the 'action observation area' (the 'pre-sensor' period from -750 ms to sensor signal) or while the grasp was maintained for a short period, released and the hand withdrawn (the 'post-sensor' period from sensor signal to + 750 ms). In the example shown in Fig. S5, in which the experimenter performed precision grip with an object, the PTN's discharge was not modulated during the 'pre-sensor' period whereas there was clear suppression of activity in the 'post-sensor' period. In seven sessions, we tested the effect of the experimenter approximately halving the speed of approach towards the food target. For 6 out of 8 mirror-like PTNs the time course of its activity was essentially unchanged. One example of such behaviour is shown in Fig. S5. The suppression of PTN discharge during slow and fast approach trials (rank-ordered for speed in Fig. S5B) were very similar

when superimposed (Fig. S5C), suggesting that this PTN was mainly responding to the final grasping action of the experimenter, not to the dynamics of the approaching movement.

Mirror like activity for non-grasp condition

In one monkey we performed additional mirror test in which, the experimenter's hand approached the food reward and covered it with her hand, without grasping it. 25% of PTNs (3/12) that showed suppression of discharge during observation of precision grip also showed suppression during these 'flat hand' actions; the other 9 PTNs were unmodulated. Similarly, 33% (3/9) of facilitation PTNs recorded during observation of precision grip were also facilitated during observation of "flat hand" actions; the other 6 PTNs were unmodulated.

References

Cattaneo, L., Fabbri-Destro, M., Boria, S., Pieraccini, C., Monti, A., Cossu, G., and Rizzolatti, G. (2007). Impairment of actions chains in autism and its possible role in intention understanding. Proc Natl Acad Sci U S A *104*, 17825-17830.

Kakei, S., Hoffman, D.S., and Strick, P.L. (2001). Direction of action is represented in the ventral premotor cortex. Nature Neuroscience *4*, 1020-1025.

Kurata, K. (1993). Premotor cortex of monkeys: set- and movement-related activity reflecting amplitude and direction of wrist movements. J Neurophysiol *69*, 187-200.

Kurata, K., and Hoshi, E. (2002). Movement-related neuronal activity reflecting the transformation of coordinates in the ventral premotor cortex of monkeys. J Neurophysiol *88*, 3118-3132.

Rizzolatti, G., Camarda, R., Fogassi, L., Gentilucci, M., Luppino, G., and Matelli, M. (1988). Functional organization of inferior area 6 in the macaque monkey. II. Area F5 and the control of distal movements. Exp Brain Res *71*, 491-507.

Umilta, M.A., Brochier, T., Spinks, R.L., and Lemon, R.N. (2007). Simultaneous recording of macaque premotor and primary motor cortex neuronal populations reveals different functional contributions to visuomotor grasp. J Neurophysiol *98*, 488-501.

Figure S1. Location of PTNs recorded in F5 and M1 for M43.

Locations of electrode penetrations in area F5 (ventral premotor cortex) of monkey M43 in which PTNs with mirror activity were encountered either with or without digit rICMS responses are shown by filled and open triangles, respectively. Circles indicate location of PTNs without mirror activity with (filled)/without (open) digit rICMS responses; the latter were obtained with current intensities of 16-60 µA. F5 PTNS were recorded in 25 separate recording sessions. Open squares indicate locations of PTNs in the hand area of M1 and recorded in the same monkey. ArcS: arcuate sulcus; CS: central sulcus. The PTNs in F5 of monkey M41 were recorded at a similar location in 4 recording sessions.

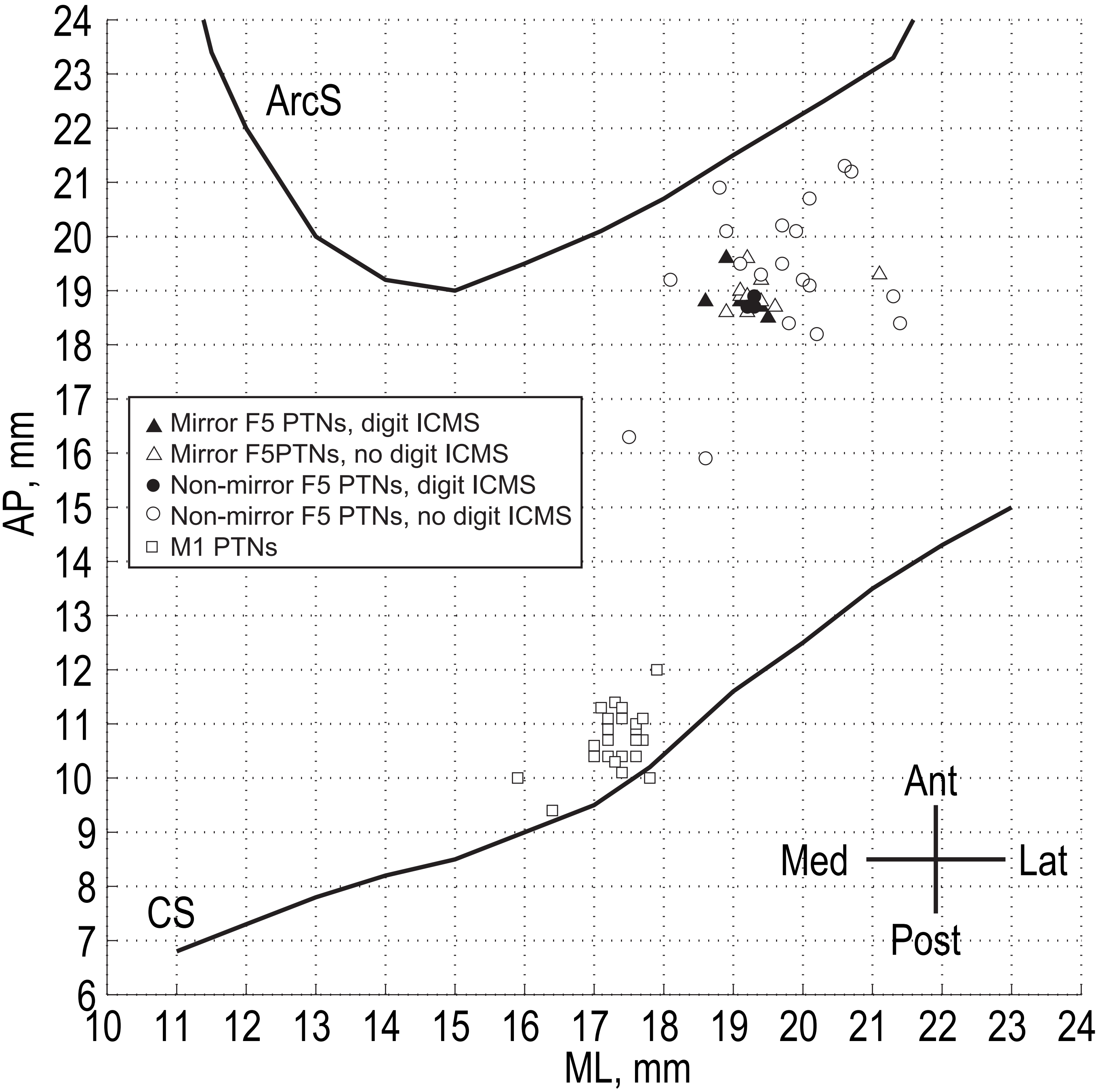
ArcS
CS
AP, mm
ML, mm
Mirror F5 PTNs, digit ICMS
Mirror F5PTNs, no digit ICMS
Non-mirror F5 PTNs, digit ICMS
Non-mirror F5 PTNs, no digit ICMS
M1 PTNs
Ant
Med
Lat
Post
24
23
22
21
20
19
18
17
16
15
14
13
12
11
10
9
8
7
6
10
11
12
13
14
15
16
17
18
19
20
21
22
23
24

Figure S2. Time course of mirror tests.

Each trial started with home pad press (HPP) by the experimenter, then after approximately 1 s the experimenter released her hand (HPR) and started movement, (shown schematically by red dashed line) towards the magnetic sensor (SP)in the action observation area. When her hand touched the sensor an event signal was generated.

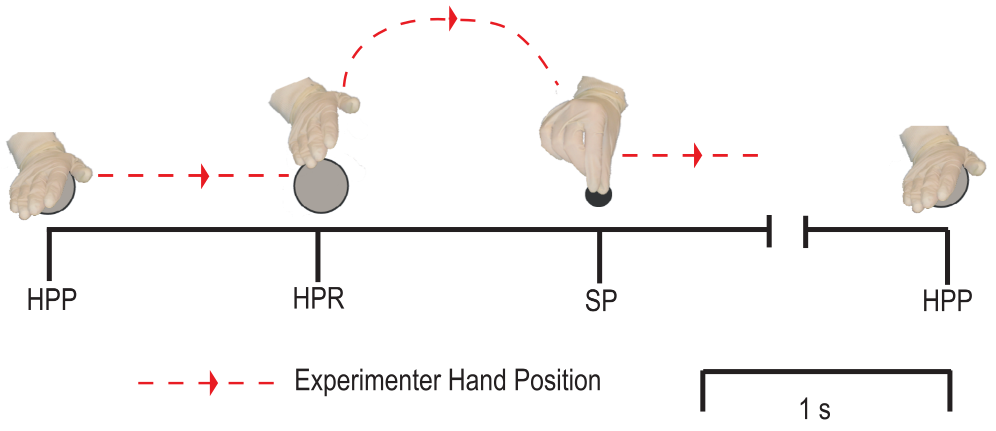
HPP
HPR
SP
HPP
Experimenter Hand Position
1 s

Figure S3. EMG control.

An example of a PTN recorded in an EMG “contaminated” session. All notations are as on Fig. 1. All trials show EMG activity from three example muscles (Biceps, ECR-L, EDC) are shown. Note the consistent EMG activity during action observation (right panel) just before the sensor touch signal at time zero. Changes in PTN discharge rate overlap with this EMG activity, which was of comparable amplitude to that recorded during active grasp (left panel).

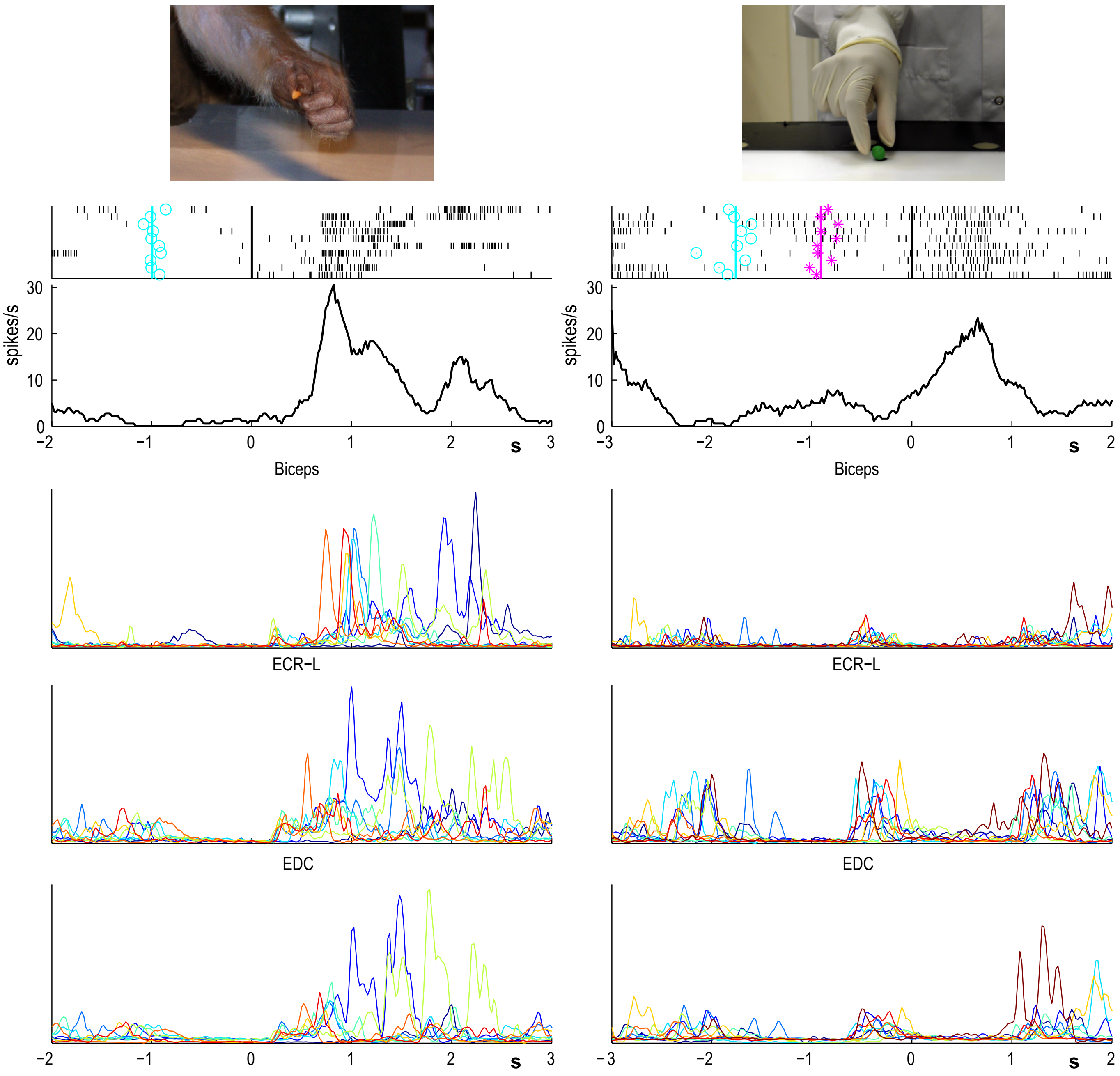
spikes/s
30
20
10
0
−2
−1
0
1
2
3
s
Biceps
ECR−L
EDC
−3
−2
−1
0
1
2

Figure S4. Control experiments

Raster plots and average firing rates of a PTN which was strongly activated when the monkey grasped with the contralateral (**A**) but not with the ipsilateral hand (**B**). Alignment is to placement of food reward by the experimenter (black vertical line at time zero) **C**: The same PTN responded during observation of precision grip with object; rasters aligned to the experimenter touching the object. Note highly significant facilitatory mirror effect.This PTN had an ADL of 0.7 ms.

D and **E** Two PTNs recorded simultaneously during observation of precision grip with object. One PTN (D) was strongly activated during this mirror test (ADL 1.8 ms) while the other (E) was not (ADL 1.2 ms).

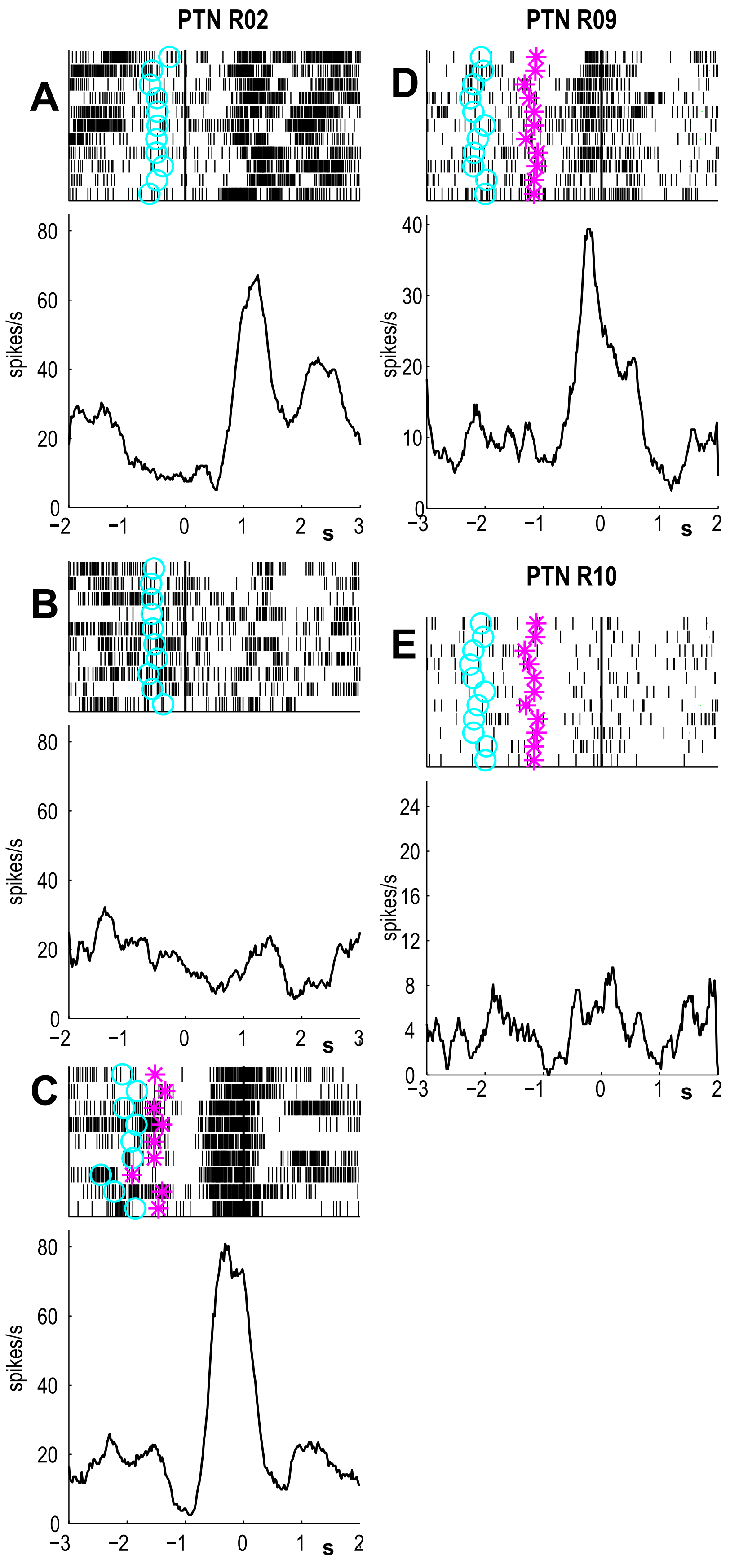
PTN R02
PTN R09
PTN R10
A
B
C
D
E
spikes/s
s
80
60
40
20
0
−2
−1
0
1
2
3
40
30
10
24
16
12
8
4
−3

Figure S5. Demonstration that mirror-like activity in a PTN is not modified by different hand transport times.

A: A raster plot and average firing rate for PTN which showed suppression during mirror testing, (precision grip plus object). **B**: Raster plot for the same PTN as in **A**, experimenter varied the hand transport time to the object; trials are ordered according to the duration of the transport time (fast trials first) of the mirror movement from the beginning (magenta asterisk) to the touch of the object (magnetic sensor signal= black vertical line). **C**: Average firing rates for raster shown in **B**, the black curve is for all 10 trials, red curve is only for fastest 5 trials, and blue curve

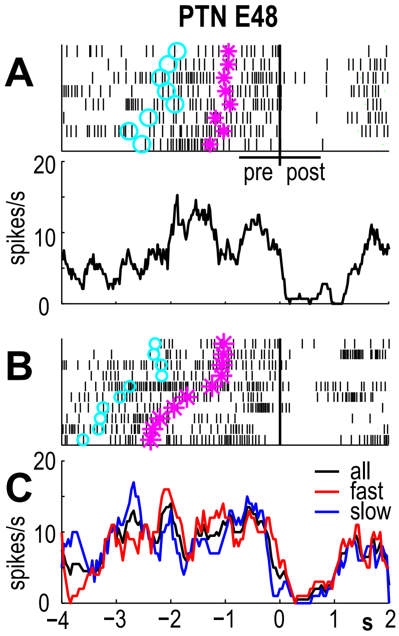
PTN E48
A
pre
post
20
spikes/s
10
0
B
C
20
all
fast
slow
spikes/s
10
0
−4
−3
−2
−1
0
1
s
2
